# Association between depressive symptoms and poor sleep quality among pregnant women in Northern Rural Bangladesh: a community-based cross-sectional study

**DOI:** 10.1186/s12888-022-03839-w

**Published:** 2022-03-19

**Authors:** Md Mahbubul Alam Shaun, Md Wahidur Rahman Nizum, Md Asaduzzaman Shuvo, Fahmida Fayeza, Md Omar Faruk, Md Fakrul Alam, Md Sabbir Ahmed, Sanjana Zaman, Sujan Kanti Mali, Mohammad Delwer Hossain Hawlader

**Affiliations:** 1 Department of Biochemistry and Food Analysis, Faculty of Nutrition and Food Science, Patuakhali Science and Technology University, Dumki 8602, Patuakhali, Bangladesh; 2 Department of Public Health, School of Health and Life Sciences, North South University, Dhaka, 1229 Bangladesh; 3 Department of Community Health and Hygiene, Faculty of Nutrition and Food Science, Patuakhali Science and Technology University, Dumki 8602, Patuakhali, Bangladesh; 4 Department of Public Health, Daffodil International University (DIU), Dhaka, 1207 Bangladesh

**Keywords:** Pregnant, Sleep quality, Depressive symptom, Northern rural area, Bangladesh

## Abstract

**Background:**

Adequate good quality of sleep is essential for physical fitness during pregnancy as well as being a depressive symptoms-free mind. However, there is little evidence of the relationship between depressive symptoms and poor sleep quality among pregnant women in Bangladesh. This study aimed to find the association between depressive symptoms and poor sleep quality among pregnant women in northern rural Bangladesh.

**Methods:**

A community-based cross-sectional study was carried out from May 2021 to June 2021 among 481 pregnant women tested positive in the pregnancy test of Jaldhaka and Dimla Upazila of Nilphamari district, Rangpur Division. Data were collected with a structured questionnaire including socio-demographic conditions, sleep quality, and depressive symptoms, comprising the Pittsburgh Sleep Quality Index (PSQI) and the Patient Health Questionnaire- 9 (PHQ-9).

**Results:**

8.94% of the women had depressive symptoms, whereas 38.88% of the participants were bad sleepers. However, women who had depressive symptoms [Adjusted odds ratio (AOR) = 2.55; 95% CI 1.33-4.9] and educational qualifications above 10 years [AOR = 0.60; 95% CI: 0.39-0.92] were associated with poor sleep quality.

**Conclusions:**

A higher percentage of pregnant women had poor sleep quality, whereas depressive symptoms and academic background of the participants were significantly associated with poor sleep quality. Ensuring adequate sleep time and better quality could be helpful to prevent depressive symptoms.

## Introduction

Sleep is an essential physiological need for human beings for normal functioning that is a natural recurred situation of both body and soul which is recognized by repeatedly changed consciousness, contracted muscle activity, relatively inhibited sensory activity, and prohibition of most voluntary muscle [1]. It is essential for the excellent state of physical and mental health as poor sleep quality and inadequate age-appropriate Total Sleep Time (TST) may adversely affect mood and wellbeing [2]. In recent years, sleep quality has become a more significant and point of concern over the TST [3]. Sleep quality may rely upon an individual's amusement of the sleep perception, sleep duration, maintenance of sleep, and refreshment after waking [4]. Sleep becomes more important during pregnancy, and it requires additional TST for a better pregnancy outcome [5]. From the beginning of conception to delivery, pregnancy and sleep have a close relationship. Sleep-related issues are prevalent among pregnant women, and it has long been recognized that sleep can be disrupted during pregnancy [6]. Hormonal changes in pregnancy affect sleep patterns, and several physiological changes during pregnancy are also known to disrupt regular sleep patterns [7, 8]. Excessive drowsiness is also a typical complaint among pregnant women, with disrupted sleep patterns beginning to shift around 11–12 weeks of pregnancy [9]. Hormonal changes connected with the reproductive cycle are now recognized as increasing the lifelong risk of emotional disorders in many women.

The gestational period is crucial for pregnant women as significant changes in a female's life like alteration in appearance, mood, and health happen in this period, leading to increased stress, anxiety, depressive symptoms, poor sleep, and impaired quality of life [10]. A study conducted at two upazillas of Mymensingh district of Bangladesh showed that 18.3% of the participants had antepartum depressive symptoms [11] whereas another research conducted at Matlab, a rural area in the Chandpur distric found that 82.2% of women reported any depressive symptoms in the last week [12]. Based on the National Sleep Foundation's statistics, in the United States of America (USA), 78% of women reported alteration in sleep, whereas another study found that 76% of women experienced a bad quality of sleep during the whole span of pregnancy [13]. Like a significant life event accompanied by hormonal changes, pregnancy can put women at a higher risk of developing or returning to depressive symptoms [14]. Poor quality of sleep is a potential risk factor for depressive symptoms both during pregnancy and after delivery (also known as postpartum depressive symptoms) [15–17]. Some women have their first depressive episode during pregnancy, while others with a history of depressive symptoms are more likely to have it recur, continue, or worsen [18]. Around 10-25% of women in Brazil were affected by depressive symptoms during pregnancy [19]. Several studies reported the adverse effect of poor sleep quality among expectant mothers. For instance, Okun, Schetter, and Glynn (2011) reported that sleep deprivation in pregnancy increased the risk of premature birth [20]. Even after controlling for depressed symptoms, Gelaye and colleagues (2015) reported that poor sleep quality during pregnancy was linked to a 1.7-fold increased risk of suicide thoughts [21].

Mental impairment of the mother and fetal neural networks is highly vulnerable to maternal sleep loss, which may occur due to poor sleep quality [22]. Altered fetal response to vibroacoustic stimulation, changed fetal heart rate variability, altered motor activity, and changed behavioral reactivity and development were identified in several studies with causes due to prenatal depressive symptoms and stress [23, 24]. However, the presence of poor sleep quality along with depressive symptoms among women during the pregnancy period could worsen both mother's and child's mental and physical health outcomes during both antenatal and postnatal periods. Previous studies also reported a higher rate of depressive symptoms among Bangladeshi pregnant women. Interestingly, even though sleep is an essential aspect of a pregnant woman's physical and mental health, little is known about the relationship between depressive symptoms and sleep quality of pregnant women. Maintenance of good sleep health during pregnancy also ignored in the antenatal care service package in Bangladesh. Therefore, it is important to work on this untouched area to explore the current scenario and bring it to the policymakers to include sleep health care components in antenatal care package for pregnant women in Bangladesh. To the best of our knowledge no study has been conducted in Bangladesh to identify the association between sleep quality and depressive symptoms among pregnant women. To fill this knowledge gap this study was aimed to identify the association between depressive symptoms and sleep quality among pregnant women of the northern rural context in Bangladesh.

## Materials and Methods

### Study design, participants, and sampling procedure

**Fig. 1:**
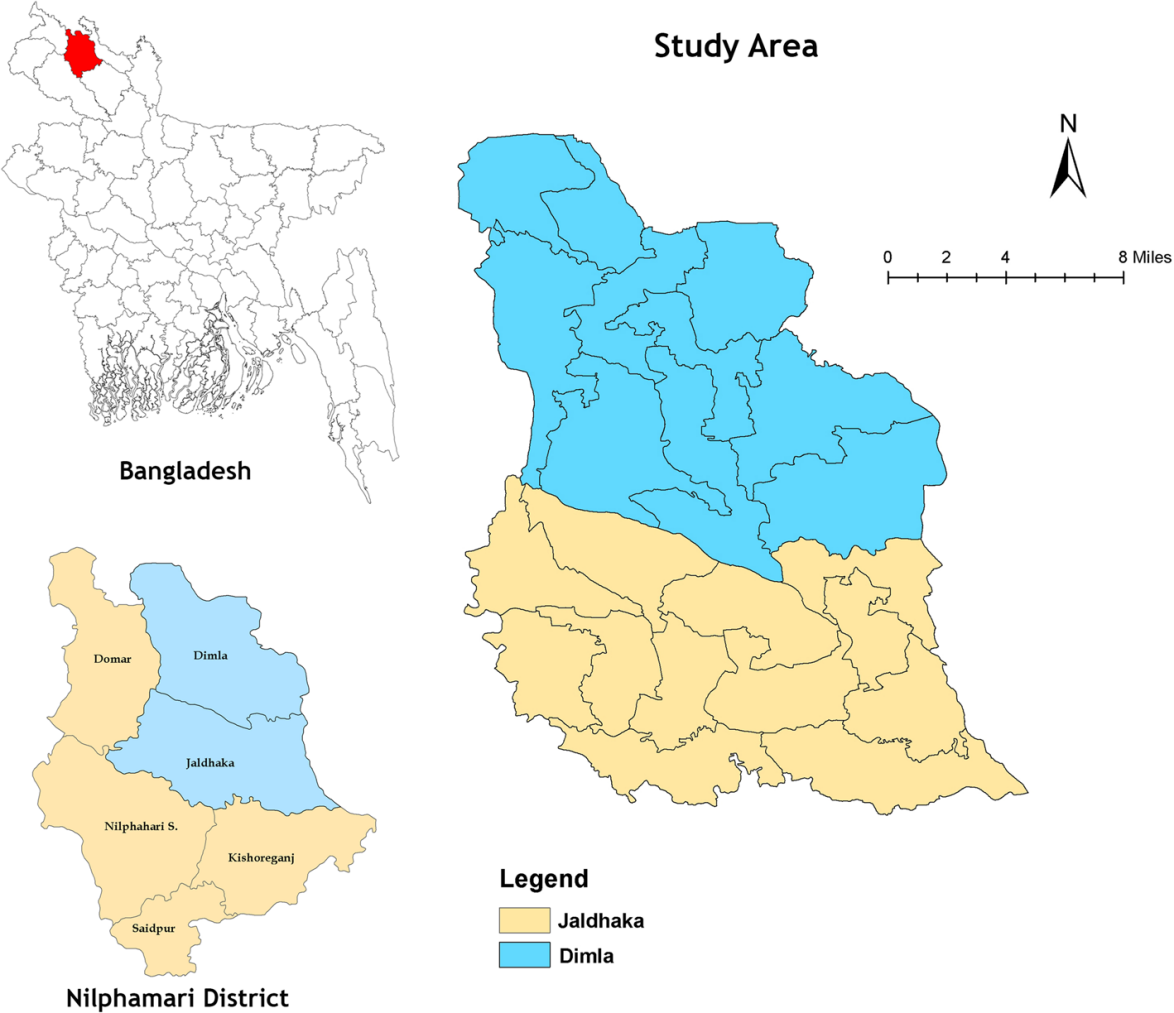
Study area

A community-based cross-sectional survey was carried out among the pregnant women of Nilphamari district, Rangpur Division, Bangladesh. The study area (Fig. 1) was Jaldhaka and Dimla Upazila of Nilphamari district. The sample size of the study was calculated following the equation of cross-sectional study for an unknown population. The calculated sample size was 384, where the confidence interval was 95% (z = 1.96), level of probability was 50% (p = 0.5) and 5% margin of error (d = 0.05). The sample size was calculated using OpenEpi (Open Source Epidemiologic Statistics for Public Health) version 3.5.4 [25]. However, data were collected from 481 pregnant women in this particular study to increase the sample size. A larger sample size gives more precision, and the significant result from a larger sample size is more acceptable than smaller studies [26]. Data were collected with a structured questionnaire, including socio-demographic conditions, sleep quality, and depressive symptoms. Before commencing final data collection, a pilot survey was conducted among 35 pregnant women outside of the study area. Women who were married, tested positive in the pregnancy test, living in the study area, and aged between 15-49 years were included in this research, whereas unmarried women not living permanently in the study region like visiting father's house were omitted from this study.

#### Data collection procedure

 A pre-structured questionnaire was used to collect data from the participants. There was three data enumerator assigned for data collection. The enumerators went to each participant's house to conduct a face-to-face interview. The authors trained the data enumerators prior to the data collection. The authors supervised the data enumerators and checked the data sheets regularly to remove errors and inconsistencies. Data enumerators took consent from the participants and read aloud the consent form for the participants who could not read in front of their family members or neighbors who could read.

### Independent variables

Socio-demographic variables along with depressive symptoms and sleep patterns were the independent variables for this study. In socio-economic characteristics, age of the participants, academic background of the participants, profession, monthly family income of the participants were independent variables, whereas, in sleep patterns, they were actual sleep time at night, time of going to bed at night, and time of wake up in the morning. However, depressive symptoms, categorized as depressive symptoms (yes/no), were the independent variable. Depressive symptoms were measured using the Patient Health Questionnaire-9 (PHQ-9) [27] that is a screening instrument with 9 items developed to measure depressive symptoms and was validated to use among pregnant women [28]. The participants were asked to assess how much they were bothered by the symptoms over the last two weeks for each item. There are four answer options: not at all (0), several days (1), more than half of the days (2), and nearly every day (3). The sum score (range 0 to 27) indicates the degree of depressive symptoms [29]. For this study, a cutoff value of ≥10 was used to define possible depressive symptoms, and cutoff value<10 was used to specify no depressive symptoms [30]. Moreover, the categories for sleep time and rise time were done based on a previous study conducted in Bangladesh [4]. For bedtime, as we all the authors from Bangladesh, experience from the usual sleep patterns, we set up the categories of bedtime.

### Outcome variable

Participant's sleep quality was the outcome variable in this particular study. The Pittsburgh Sleep Quality Index (PSQI) [30], an 18-item questionnaire, measured habitual sleep quality over the previous month. It comprises 7 subscales assessing subjective sleep quality, sleep latency, sleep duration, habitual sleep efficiency, sleep disturbances, use of sleeping medication, and daytime dysfunction. Each subscale has a possible score of between 0 and 3, with an overall global score of 0–21. Higher scores reflect poorer sleep quality. Participants were divided into two groups for analysis: good sleep quality (score <5) and poor sleep quality (score ≥5) [4].

### Data analysis

Data were cleaned, coded, and edited before commencing formal analysis. All the statistical analyses were conducted by Stata 16.0 (Stata Corp, College Station, TX, USA) software. Descriptive analysis was conducted to determine the frequency and percentage of the socio-demographic variables, components of the PSQI scale, and depressive symptoms among the respondents. A Chi-square test was conducted to assess the association between the outcome variable (sleep quality) and depressive symptoms and socio-economic variables. Binary logistic regression was run to determine whether sleep quality is associated with depressive symptoms and other independent variables. An entry method of binary logistic regression was done for the effect of depression on sleep quality, adjusting only those variables found p-value <0.250 in the Chi-square test. We tried our best to consider all possible confounders, that’s why we broaden the p-value <0.250 instead of p-value <0.05.The level of significance was set at 5% for binary logistic regression.

## Results

### Socio-demographic characteristics

Table 1 represents the socio-demographic status and the results of the Chi-square test. The average age of the participants was 24.4 years (SD = ±4.1), 50.21% of the participants were aged 25 years or above, whereas the rests were below 25 years.38.05% of the participants had above 10 years of educational qualification. Among the 481 participants, almost all of them were housewives (91.0%), and nearly two-thirds (61.54%) of the participants had a monthly income less than Bangladeshi Taka (BDT) 10000. About two-thirds (69.0%) of the surveyed population had more than 7 hours of sleep at night, while 91.06% of the participants showed no depressive symptoms. Just below half of the population (43.66%) used to go to bed between 10:00-10:59 PM, and the almost identical number of participants (44.0%) woke up between 06:00-06:59 AM. Only 8.94% of the women had depressive symptoms (PHQ-9 cutoff value ≥ 10). The prevalence of poor sleep quality was high among the participants who had 6 to 10 years of education (52.54%; *p* = 0.003), and who had depressive symptoms (58.14%; *p* = 0.007).

**Table 1:** Socio-demographic characteristics and distribution of sleep quality against them

Variables	Frequency (%)	Quality of sleep (%)		x2 df	***p*** Value
		Good	Poor		
**Age of the participants**^**a**^	Mean (SD) 24.4 (±4.1)			2.06	0.151
Below 25 years	236 (49.79)	152 (64.1)	84 (35.59)		
25 years and above	238 (50.21)	138 (57.98)	100 (42.02)		
**Education of the participants**					
0 to 5 years	239 (49.69)	138 (57.74)	101 (42.26)	11.78	**0.003**
6 to 10 years	59 (12.27)	28 (47.46)	31 (52.54)		
Above 10 years	183 (38.05)	128 (69.95)	55 (30.05)		
**Occupation of the participants**					
Housewife	438 (91.06)	273 (62.33)	165 (37.67)	3.03	0.220
Employee	24 (4.99)	12 (50.00)	12 (50.00)		
Business and others	19 (3.95)	9 (47.37)	10 (52.63)		
**Monthly Household Income (BDT**^**b**^**)**					
Below 10000	296 (61.54)	177 (59.8)	119 (40.2)	0.83	0.662
10000-15000	132 (27.44)	85 (64.39)	47 (35.61)		
Above 15000	53 (11.02)	32 (60.38)	21 (39.62)		
**Actual Sleep time at night**					
<7 Hours	149 (30.98)	86 (57.72)	63 (42.28)	1.05	0.305
≥7 hours	332 (69.02)	208 (62.65)	124 (37.35)		
**Depressive symptoms**					
No	438 (91.06)	276 (63.01)	162 (36.99)	7.37	**0.007**
Yes	43 (8.94)	18 (41.86)	25 (58.14)		
**Time of going to bed at night**					
Before 09:00 PM	61 (12.68)	41 (67.21)	20 (32.79)	1.44	0.697
09:00-09:59 PM	160 (33.26)	96 (60.00)	64 (40.00)		
10:00-10:59 PM	210 (43.66)	125 (59.52)	85 (40.48)		
After 11:00 PM	50 (10.4)	32 (64.00)	18 (36.00)		
**Time of getting up in the morning**					
Before 06:00 AM	114 (23.7)	71 (62.28)	43 (37.72)	1.06	0.788
06:00-06:59 AM	212 (44.07)	125 (58.96)	87 (41.04)		
07:00-07:59 AM	130 (27.03)	81 (62.31)	49 (37.69)		
After 08:00 AM	25 (5.2)	17 (68.00)	8 (32.00)		

^a^ Age has seven missing values

^b^*BDT *Bangladeshi Taka; 10000 BDT= 118 USD

### Sleep quality and its component scores among study participants

The following table 2 represents the PSQI components of the study population. Among the 481 participants, about one-third (38.88%) of the respondent had poor sleep quality (PSQI score >5). The global mean PSQI score was 4.4 (SD= ±2.0) among pregnant women. About 72% of the participants described their sleep quality as fairly good. Almost half of the participants (48.86%) had sleep latency of 16-30 minutes, and habitual sleep efficiency was more than 85% among 76.51% of the respondents. 74.43% of the study population had more than 7 hours of sleep at night, and 89.81% had never used sleeping pills during their pregnancy. About 76.92% of the pregnant women had little disturbance (1 to 9) while sleeping, and as a result, 48.44% of them had little daytime dysfunction (1 to 2).

**Table 2:** Sleep quality and its component scores among study participants

Components	Frequency	Percent
PSQI Score mean (±SD)	4.4 (SD= ±2.0)	
**Subjective sleep quality**		
Very good	109	22.66
Fairly good	349	72.56
Fairly bad	19	3.95
Very bad	4	0.83
**Sleep latency**		
0	188	39.09
16-30 min	235	48.86
31-60 minutes	48	9.98
>60 minutes	10	2.08
**Sleep duration**		
More than 7 hours	358	74.43
6-7 hours	102	21.21
5-6 hours	19	3.95
Less than 5 hours	2	0.42
**Habitual sleep efficiency**		
>85%	368	76.51
75-84%	39	8.11
65-74%	60	12.47
<65%	14	2.91
**Sleep disturbances**		
0	45	9.36
1 to 9	370	76.92
10 to 18	62	12.89
19 to 27	4	0.83
**Use of sleeping medications**		
Never	432	89.81
Less than once a week	30	6.24
1-2 times a week	14	2.91
3 or more than 3 times a week	5	1.04
**Daytime dysfunction**		
0	157	32.64
1 to 2	233	48.44
3 to 4	85	17.67
5 to 6	6	1.25

### Association among socio-economic characteristics, depressive symptoms, and poor sleep quality

Below table 3 illustrates the relationship among socioeconomic characteristics, depressive symptoms, and poor sleep quality of pregnant women. Women who had depressive symptoms were 2.5 times higher for poor sleep quality than those who had no depressive symptoms [Adjusted odds ratio (AOR) = 2.55; 95% CI: 1.33-4.90]. However, the odds were 0.60 times lower among the women who had above 10 years of schooling compared to their counterparts [AOR = 0.60; 95% CI: 0.39-0.92].

**Table 3:** Bivariate and multiple logistic regression model of factors independently associated with poor sleep quality among pregnant mothers

Variables	Unadjusted model			Adjusted Model		
	Odds Ratio	*p* value	95% Confidence Interval	*Odds Ratio*	*p* value	95% Confidence Interval
**Participants having depressive symptoms**						
No	Ref			Ref		
Yes	2.37	0.008	1.25-4.47	2.55	**0.005**	1.33-4.9
**Age of the participants**						
Below 25 years	Ref			Ref		
25 years and above	1.31	0.152	0.91-1.9	1.43	0.068	0.97-2.1
**Education of the participants**						
0 to 5 years	Ref			Ref		
6 to 10 years	1.51	0.156	0.85-2.68	1.62	0.119	0.84-2.99
Above 10 years	0.59	0.01	0.39-0.88	0.60	**0.018**	0.39-0.92
**Occupation of the participants**						
Housewife	Ref			Ref		
Employee	1.65	0.231	0.73-3.77	2.00	0.119	0.84-4.82
Business and Others	1.84	0.195	0.73-4.62	1.84	0.233	0.67-5.04

## Discussion

This study found a significant positive association between depressive symptoms and poor sleep quality among pregnant mothers in Bangladesh. Having sleep disorders and depressive symptoms can cause premature birth [31]. Sleep problems during pregnancy may be connected to unfavorable outcomes like gestational hypertension and cesarean delivery [32]. Moreover, birth type, labor pain duration, baby weight, Apgar score can be affected by the quality of sleep and sleep duration [33]. About one-third of the studied population had poor sleep quality; just nearly about 10% had depressive symptoms. Furthermore, we have found that academic qualifications are also significantly associated with poor sleep quality.

Overall, one-third (38.88%) of the women had poor sleep quality in our study. A similar result was found in Vietnam (41.2%) [34]. However, significantly higher prevalence of poor sleep quality were found in other studies in Ethiopia (68.4%) [5], Malaysia (69.4%) [35], the USA (76%) [36], Brazil (56.3%) [37], and Pakistan (53.3%) [38]. Plausible reasons behind these differences may be due to the study design, sample size, and participants' socio-economic status. Moreover, the difference may be explained as only healthy pregnant women were included in our study. However, poor sleep quality develops due to many factors during the pregnancy period. For instance, having depressive symptoms, anxiety, lower academic qualifications, higher age of respondents, and late gestational age were found significantly associated determinants to have poor sleep quality among the pregnant mothers in several studies [5, 31, 39]. Poor sleep quality may bring about the mother's mental impairment, diabetes during gestation, preterm birth, depressive symptoms, placental abruption, small for gestational age, and cesarean delivery, and the offspring are predisposed to various anxiety disorders and learning disabilities [40]. Therefore, pregnant mothers need health education about poor sleep quality and sleep hygiene practice risk factors.

We have found that 8.94% of the women had depressive symptoms. Other studies show depressive symptoms among the higher proportion of pregnant women in Brazil [41], India [42], and Ethiopia [43–45]. The primary cause of developing depressive symptoms during pregnancy is irregular changes in hormones [46]. However, many more socio-economic factors are significantly related to form depressive symptoms among pregnant women were shown in several studies. The most common independent determinants are the educational level of the pregnant women and their husbands, having an undesired pregnancy, suffering from a chronic disease before pregnancy, presence of pregnancy-related problems, having a child with a disability or having relatives whose children had a disability, smoking during pregnancy, past personal or family history of depressive symptoms, single marital status, poor health functioning, maternal anxiety, life stress, lack of social support, domestic violence, lower-income, and poor relationship quality with the partner [47–52]. Though the prevalence of depressive symptoms in our study area is just below worldwide prevalence (10-20%) [43], special care should be taken to minimize it by increasing rest time and sleep time support from husband and other family members. This particular study revealed that depressive symptoms and poor sleep quality have a significant relationship as women having depressive symptoms are more likely to have poor sleep quality. It was in line with other studies among pregnant mothers in Australia [53], in the United Kingdom [54], in the USA [55], and in China [56, 57]**.** In previous studies, a significant association between depressive symptoms and sleep quality was found, suggesting depressive symptoms as the psychological issue that contributed to poor sleep quality [58–61]. Sleeplessness creates depressive symptoms and a poor mental health state [62–64]. Sleep quality also become worsens due to a higher prevalence of depressive symptoms [65]. Moreover, sleep problems' effects on the pathogenesis of depressive symptoms were found in both epidemiologic and electroencephalographic sleep studies [62, 66, 67].

Several past studies revealed that an increase in age is significantly associated with poor sleep quality [5, 68, 69]. Sleep quality decreased due to the influence of physiological and psychological factors among older than younger pregnant women [5, 70]. However, we 'didn't find any significant association between participants' age and poor sleep quality. More advanced and compact studies might be needed to find out the plausible reasons behind this.

We have found that pregnant ' 'women's education is negatively associated with poor sleep quality. Those who completed 10 or more years of schooling were less likely to have poor sleep quality. The pathology behind this association is not clear to us. A study shows that lower academic qualified pregnant were poor sleepers [39]. However, a previous study showed no association between academic background and bad sleep quality [68]. Although we 'don't have enough evidence but this association can be explained by the fact that higher educational attainment may enable pregnant women to better take care of themselves during pregnancy and have adequate time for rest and total sleep time. However, a future mixed-method study is warranted to explore the relationship between sleep quality and educational status of pregnant women for possible intervention design.

### Strengths, Limitations, and Further Scopes

The main strength of our study is that we have used PSQI and PHQ-9 questionnaires to assess sleep quality and depressive symptoms, which are validated in the various previous studies. Moreover, we have conducted the survey among a large population which is another strength of our research, and we also conducted a pilot test before conducting the study. However, we still have some limitations. We have conducted a cross-sectional study that cannot conclude causality between any variables studied. Furthermore, we did not count the trimesters and sleep apnea due to body weight gain as previous researches showed the prevalence of bad sleep quality and depressive symptoms are higher at later days of pregnancy. We also did not count some socio-cultural factors like the women's living house belonging to the husband or father, the number of children, the presence of disabled persons within the family, gestational age, social relationship, family violence, marital status, history of alcohol consumption, previous history of depressive symptoms, comorbidity, number of pregnancies, and any caring responsibilities that may affect our findings. However, as a pioneer study, this research would be useful for future research in another region and another context to identify the relationship between depressive symptoms and poor sleep quality and plausible causes behind developing depressive symptoms and sleep disturbances, which will eventually be impactful in implementing proper interventions develop good mental health.

## Conclusions

This particular study concluded that just over one-third of pregnant women suffer from poor sleep quality. Having depressive symptoms and increased age are the most common determinants of poor sleep quality, whereas women's higher education can protect it. Since these factors are determinants of sleep quality in pregnant women, further research should incorporate these variables into a supportive model of sleep-related antenatal care. Family members should be supportive and caring enough during pregnancy to lessen the problems.

## Data Availability

Data will be available upon request from the corresponding author.

## Abbreviations

TST: Total Sleep Time
PHQ-9: Patient Health Questionnaire-9
PSQI: Pittsburgh Sleep Quality Index
SD: Standard Deviation
BDT: Bangladeshi Taka
AOR: Adjusted odds ratio
CI: Confidence Interval
USA: United States of America
